# Asking questions of psychedelic microdosing

**DOI:** 10.7554/eLife.66920

**Published:** 2021-03-02

**Authors:** Lindsay P Cameron

**Affiliations:** Graduate Program in Neuroscience, University of California, DavisDavisUnited States

**Keywords:** psychedelics, placebo, microdosing, self-blinding, expectations, citizen science, Human

## Abstract

A citizen science approach to research has shown that the improvements in mood and cognition associated with psychedelic microdosing are likely due to a placebo effect.

**Related research article** Szigeti B, Kaertner L, Blemings A, Rosas F, Feilding A, Nutt DJ, Carhart-Harris RL, Erritzoe D. 2021. Self-blinding citizen science to explore psychedelic microdosing. *eLife* **10**:e62878. doi: 10.7554/eLife.62878

What if a drug could make you smarter? Enhance creativity? Treat depression and anxiety while improving cognitive performance? These are all claims that have been made about psychedelic microdosing (Anderson et al., 2019; Johnstad, 2018). This practice involves taking sub-hallucinogenic doses of psychedelic compounds – usually LSD or psilocybin – on an intermittent basis, generally two or three times per week and almost always without medical supervision or guidance (Fadiman, 2011). Doses are typically one tenth of the amount used in a regular ‘trip’, allowing individuals to function normally after taking the drug. Microdosing first gained popularity in places like Silicon Valley for its purported effects and has spread rapidly across the globe.

A few studies have investigated the therapeutic effects of microdosing, but many of these have been observational studies in which most data were gathered over internet platforms. Individuals who microdose tend to report improvements in depression, anxiety, sociability, creativity and general cognition, but these studies are wrought with confounding factors, including subjects who are both self-medicating and self-reporting (Anderson et al., 2019; Cameron et al., 2020; Prochazkova et al., 2018; Polito and Stevenson, 2019). In addition, participants also report using a wide range of drugs, which vary in purity and dose (Kuypers et al., 2019). Controlled studies with illicit substances are challenging to conduct, and thus are few in number. These studies usually assess the effects of a single sub-hallucinogenic dose of a psychedelic in healthy individuals (Yanakieva et al., 2019; Bershad et al., 2019; Hutten et al., 2020). While more stringently designed, such studies often have small sample sizes and lack a placebo control group. Moreover, these controlled studies do not reflect the dosing practice of taking these drugs on a chronic, intermittent basis.

Now, in eLife, Balász Szigeti (Imperial College) and colleagues report how they have taken a citizen science approach to enroll 191 participants in a trial, and then used a clever experimental protocol to blind these participants to the experimental conditions (Szigeti et al., 2021). Participants were split into three groups and took doses for four weeks: the first group microdosed, the second group took only placebo, and the last group had two weeks of microdoses and two weeks of placebo (Figure 1).

**Figure 1.**
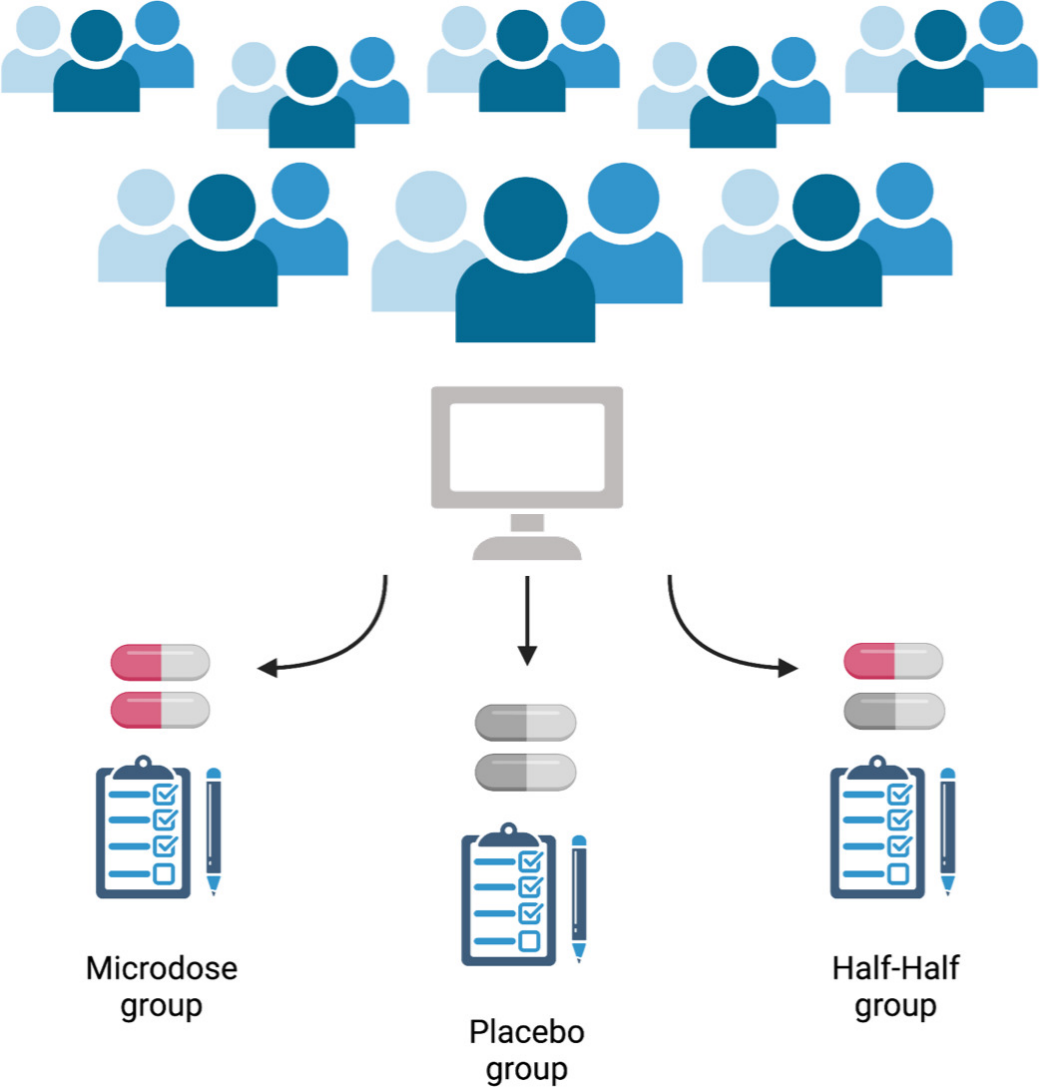
Schematic of the self-blinding microdosing experiment. Participants signed up to the study on https://selfblinding-microdose.org/ (top), and were provided with instructions on how to perform the experiments. Szigeti et al. sent the participants opaque capsules, zip bags, envelopes and QR codes. First, the participants prepared microdoses by placing drugs into the opaque capsules. Empty capsules were used as placebos. Once the microdoses and placebos were prepared, sets of capsules for each week of the trial were assembled according to the dose schedule, and each capsule was placed in a zip bag with a label indicating what day of the week it should be taken. Every participant prepared eight sets of weekly capsules, four ‘microdose’ sets and four ‘placebo’ sets (participants had to be unaware of whether they were taking the placebo, the drug, or a half-dose, so it was important that they prepare for any of the three regimes). Each weekly set of capsules was then placed into an envelope along with a single QR code that identified whether the envelope contained placebo or microdose capsules (the QR code was used by Szigeti et al. afterwards to determine what each participant had taken). These envelopes were then placed in pairs into four big envelopes. Each big envelope contained either two microdose sets of capsules or two placebo sets of capsules. The big envelopes were then shuffled and two were chosen using a semi-randomized drawing process. The other two big envelopes were discarded. The drawing process was designed so that each participant would have a one in three chance of drawing envelopes matching one of the three possible regimes (bottom): either a full microdose regime (both big envelopes contained microdose weekly sets), a half microdose regime (one big envelope contained microdose weekly sets and one contained placebo weekly sets), or a placebo regime (both big envelopes contained placebo weekly sets). This resulted in three experimental groups of approximately the same number of participants following each regime, without the participants themselves knowing what they were taking. This approach to setting up the study allowed participants to blind themselves to what they were taking, while at the same time overcoming the financial and regulatory hurdles associated with drug studies.

Surveys were given to participants at the start of the study, at multiple points during the investigation, and afterwards to measure a wide range of psychological outcomes including creativity, emotional state, mood, energy, well-being, mindfulness, openness, neuroticism and paranoia. Critically, their method enabled a placebo-controlled study, with a large sample size and realistic drug-use practices (albeit with drug samples that vary in purity and dose). This is the largest placebo-controlled microdosing study to date.

While Szigeti et al. confirm anecdotal reports that microdosing improves mood and cognitive functions, there was no significant difference between the microdosing group and the placebo-treated group. This suggests that effects associated with psychedelic microdosing can be explained by the placebo effect. Consistent with this, participants scored significantly higher on the surveys when they believed they had taken a microdose.

So, does the dose of a psychedelic compound have to be strong enough to cause hallucinations in order to have a therapeutic effect? The results of Szigeti et al. suggest that the answer to this question is yes. However, as in many other placebo-controlled trials, the participants in the latest study had to have previously used psychedelics. This may confound the results of the current study as the therapeutic effects of a single fully hallucinogenic dose can last for several years (Agin-Liebes et al., 2020).

Importantly, participants in this study were primarily healthy individuals with few reporting a diagnosed psychiatric condition. Other studies have demonstrated that clinical populations with diagnosed psychiatric disorders exhibit a more robust response to treatment. In this light, there may be a role for psychedelic microdosing in individuals with a psychiatric diagnosis, particularly if they have never taken drugs.

Finally, in addition to demonstrating that the improvement in mood and cognitive function caused by microdosing may be due to the placebo effect, Szigeti et al. have taken a significant step forward in the field of psychedelics by showing how citizen science approaches can be used to conduct large, placebo-controlled studies.
